# Current Concepts of Neural Stem/Progenitor Cell Therapy for Chronic Spinal Cord Injury

**DOI:** 10.3389/fncel.2021.794692

**Published:** 2022-02-03

**Authors:** Hidenori Suzuki, Yasuaki Imajo, Masahiro Funaba, Norihiro Nishida, Takuya Sakamoto, Takashi Sakai

**Affiliations:** Department of Orthopedic Surgery, Graduate School of Medicine, Yamaguchi University, Ube, Japan

**Keywords:** chronic spinal cord injury, neural stem/progenitor cell, glial scar, chondroitin sulfate proteoglycans, regenerative medicine

## Abstract

Chronic spinal cord injury (SCI) is a devastating condition that results in major neurological deficits and social burden. It continues to be managed symptomatically, and no real therapeutic strategies have been devised for its treatment. Neural stem/neural progenitor cells (NSCs/NPCs) being used for the treatment of chronic SCI in experimental SCI models can not only replace the lost cells and remyelinate axons in the injury site but also support their growth and provide neuroprotective factors. Currently, several clinical studies using NSCs/NPCs are underway worldwide. NSCs/NPCs also have the potential to differentiate into all three neuroglial lineages to regenerate neural circuits, demyelinate denuded axons, and provide trophic support to endogenous cells. This article explains the challenging pathophysiology of chronic SCI and discusses key NSC/NPC-based techniques having the greatest potential for translation over the next decade.

## Highlights

- The lack of repair following chronic SCI is a result of intrinsic neuronal cell factors and the extrinsic SCI environment.
- NSCs/NPCs exhibit a promising therapeutic strategy to complement clinical practice by replacing the three neuronal cell types; neurons, oligodendrocytes and astrocytes that are lost after SCI.
- The two main aims of NSCs/NPCs-based treatment for chronic SCI are replacing lost cells such as neurons and oligodendrocytes and providing the cells with a microenvironment that supports or enhances the ability of cells within a lesion to provide neuroprotection and promote regeneration.
- Chondroitin Sulfate Proteoglycans (CSPGs) are recognized mostly inhibitory effects and can hinder regeneration of axons across lesions in chronic SCI environment. Chondroitinase ABC (ChABC) is a bacterial enzyme that can effectively degrade CSPGs. ChABC pretreatment can ‘unlock’ the chronically injured spinal cord to produce a microenvironment conducive to regenerative NSCs/NPCs therapy
- NSCs/NPCs treatment has the promising attempts in treatment for chronic SCI from the previous preclinical trials. However there are still critical points in clinical studies.
- Several clinical trials are ongoing using NSCs/NPCs treatment in chronic SCI.

## Introduction

Severe traumatic spinal cord injury (SCI) disrupts long descending and ascending nerve fibers as well as the orientated glial framework of white matter tracts, thus causing a loss of motor, sensory, and autonomic function. The subsequent formation of reactive tissue scarring and cystic cavitation results in the development of molecular and physical barriers to regenerative axonal growth as well as long term neurological deficits in chronic SCI (Adams and Cavanagh, 2004; Singh et al., 2014; Ahuja and Fehlings, 2016; Ahuja et al., 2017; Angeli et al., 2018). It is estimated that worldwide, SCI effects from 250,000 to 500,000 people per year (Singh et al., 2014). Thanks to programs designed to prevent the debilitating long-term effects of SCI, many of these affected individuals remain healthy and productive. While these advances in care are dramatic, there remains a pressing need for treatments that can improve repair processes and recovery in individuals with longstanding SCI. Despite extensive research, no effective treatment has been developed to repair chronic SCI (Houle and Tessler, 2003; Bareyre et al., 2004; Armour et al., 2016; Badhiwala et al., 2018; Ashammakhi et al., 2019).

Various cell populations can be used for the treatment of chronic SCI in experimental SCI models (Steeves et al., 2007; Courtine et al., 2008; Blesch and Tuszynski, 2009; Hejcl et al., 2010; Ruff et al., 2012; Amr et al., 2014; Kadoya et al., 2016; Suzuki et al., 2017; Zhao et al., 2017; Nori et al., 2018; Courtine and Sofroniew, 2019; Ruzicka et al., 2019). Several clinical trials using stem cells are currently underway around the world (Fawcett et al., 2007^1^; accessed 20 August 2021). Among these trials, therapies using exogenous neural stem cells (NSCs) appear to be particularly promising because of the ability of these cells to differentiate into all three neuroglial lineages to allow regeneration of neural circuits, demyelination of denuded axons, and trophic support of endogenous cells (Karimi-Abdolrezaee et al., 2006, 2010; Ruff et al., 2012; Kadoya et al., 2016; Suzuki et al., 2017).

This article explains the challenging pathophysiology of chronic SCI and discusses key neural stem cell and neural progenitor cell (NSC/NPC)-based techniques having the greatest potential for translation over the next decade.

## Pathophysiology of Chronic Spinal Cord Injury

**FIGURE 1:**
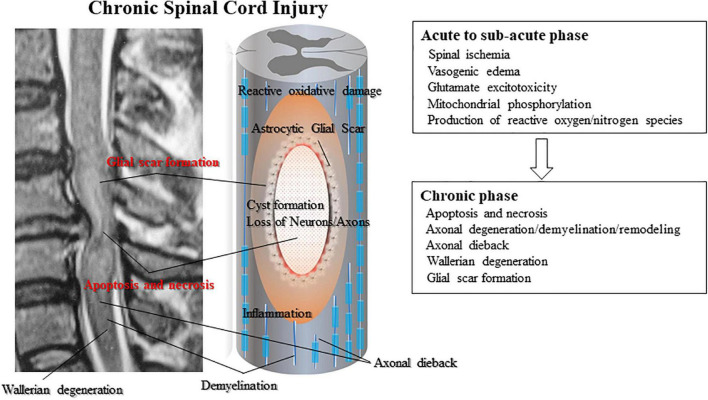
Subsequent secondary injury is characterized by further neuronal/axonal cell death and myelin degradation led by secondary inflammation from infiltrating lymphocytes and monocytes. Activated astrocytes composing the glial scar secret reactive oxygen species that widen the damaged area of the spinal cord. Glial scarring and post-traumatic cyst or syrinx formation create physical impediments to regeneration. Neurons are further damaged by post-traumatic cyst formation that exerts physical pressure on the damaged axons.

The primary injury triggers secondary injury in SCI. Secondary injury produces further chemical and mechanical damage to spinal tissues; leads to neuronal excitotoxicity caused by hemorrhage, high calcium accumulation, and enzymatic lipid hydrolysis; and increases reactive oxygen concentrations and glutamate levels (Tator and Fehlings, 1991; Proskuryakov et al., 2003; Ruff et al., 2012). Clinical manifestations of secondary injury include increased cell permeability, apoptotic signaling, ischemia, vascular damage, edema, excitotoxicity, ionic deregulation, inflammation, lipid peroxidation, free radical formation, demyelination, Wallerian degeneration, fibro-glial scarring, and cystic formation as shown in Figure 1 (de Leon et al., 1999; Fleming et al., 2006; Beck et al., 2010; Austin et al., 2012; Ruff et al., 2012; Anderson et al., 2016; Anwar et al., 2016; Badner et al., 2016; Hayashi et al., 2018).

Apoptosis is a morphologically defined type of programmed cell death that occurs in various different circumstances such as immune cell selection, carcinogenesis, and the development of necrosis (Proskuryakov et al., 2003). Typical posttraumatic necrosis occurs after SCI. Apoptotic cells have been found from 6 h to 3 weeks after injury, primarily in the spinal white matter (Ruff et al., 2012) and also within remote degenerating fiber tracts. Apoptosis appears to at least partially cause secondary degeneration occurring at the site of SCI and chronic demyelination of tracts leading away from the injury (Alizadeh et al., 2015; Ahuja and Fehlings, 2016; Anderson et al., 2016; Ahuja et al., 2017).

A unique feature of the pathological change after SCI is the progressive enlargement of the lesion area, which usually results in cavity formation and is accompanied by reactive astrogliosis and chronic inflammation (Anwar et al., 2016; Assinck et al., 2017; Yang et al., 2020). Reactive astrocytes line the spinal cavity to wall off the lesion core from normal spinal tissue (Barnabé-Heider et al., 2010; Ruff et al., 2012; Beattie and Hippenmeyer, 2017).

The lack of repair following SCI is a result of intrinsic cell factors and the extrinsic injury environment (Bradbury et al., 2002; Burda and Sofroniew, 2014; Bradbury and Burnside, 2019). To unlock the regenerative potential at the cell body level of the neuron, experimental efforts have focused on growth signaling pathways, individual genes associated with regeneration, and the transcriptional and epigenetic network (Burnside et al., 2018; Bradbury and Burnside, 2019). Scar formation also plays a key role in limiting regeneration. The scar acts to spatially contain and isolate the damage, but additional gliotic scar formation, the development of cysts and syrinxes inside the lesion, and continuous Wallerian degeneration of the severed and injured axons are the main characteristics in the chronic stage (Andrews et al., 2012; Avram et al., 2014). Unfortunately, the application of stem cells at the chronic stage has not been reported to result in any clinically significant functional improvement.

## American Spinal Cord Injury Association Impairment Scale

In clinical trials in chronic SCI, the methods of the measurement of neurological recovery is most important issues. The most standard measurement is American Spinal Cord Injury Association (ASIA) Impairment Scale. The International Standards for Neurological Classification of Spinal Cord Injury, commonly referred to as the ASIA Exam, was developed by the ASIA as a universal classification tool for spinal cord injuries based on a standardized sensory and motor assessment (Asia and ISCoS International Standards Committee, 2019). In clinical trials in chronic SCI, this scale is the most important assessment tool of recovery. The following ASIA Impairment Scale (AIS) designation is used in grading the degree of impairment (Table 1).

**TABLE 1:** American Spinal Cord Injury Association impairment scale (AIS).

Grade	Type of Injury	Description of injury
A	Complete	No Sensory or Motor Function is preserved in the Sacral Segments S4-S5
B	Sensory incomplete	Sensory but not Motor Function is preserved below the neurological level and includes the Sacral Segments S4-S5, and No Motor Function is preserved more than three levels below the Motor Level on either side of the body
C	Motor incomplete	Motor Function is preserved below the Neurological Level and More than half of key muscle functions below the Neurological Level of Injury have a muscle grade less than 3 (Grades 0–2)
D	Motor incomplete	Motor Function is preserved below the neurological level and At least half (half or more) of key muscle functions below the NLI have a muscle grade ≥ 3
E	Normal	If sensation and motor function as tested with the ISNCSCI are graded as normal in all segments and the patient had prior deficits Then the AIS Grade is E.

The scale involves both a motor and sensory examination to determine the sensory level and motor level for each side of the body, the single neurological level of injury and whether the injury is complete and incomplete (Burns et al., 2012).

## Chondroitin Sulfate Proteoglycans and Chondroitinase ABC

As a class of extracellular matrix molecule proteoglycans, chondroitin sulfate proteoglycans (CSPGs) are widely expressed within the central nervous system (CNS) and can be synthesized by all neural cell types (Andrews et al., 2012; Avram et al., 2014; Alizadeh et al., 2015). Scar tissues produce many kinds of extracellular matrix components with growth-promoting properties, fibronectin and laminin, indicating possible repairing role of astrogliosis after CNS damage (Silver and Miller, 2004). In early phase, astrogliosis is a defense response of CNS to minimize and repair primary damage, including isolation of intact tissue from secondary lesion, maintenance of favorable environment for surviving neurons and generation of permissive substrates for neurite elongation (Karimi-Abdolrezaee et al., 2010).

However, after injury to the nervous system, high upregulation of CSPGs occurs in the glial scar. In addition, CSPGs exert mostly inhibitory effects and can hinder regeneration of axons across lesions in chronic SCI (Avram et al., 2014; Silver and Silver, 2014). Despite these reports, some article revealed that tissues that strongly express CSPGs do not always exclude the entry of axons (Oakley and Tosney, 1991; Yaginuma and Oppenheim, 1991; Bandtlow and Zimmermann, 2000). In other papers reported that CSPGs coincides with developing axon pathways (Sheppard et al., 1991; Bicknese et al., 1994). Furthermore, several studies suggest that CSPG promote rather than inhibit neurite outgrowth (Streit et al., 1993).

It is still controversial whether scar-forming astrocytes are primary producing CSPGs. Some articles reported that ablating CSPG-producing cell types, astrocytes and NG2-OPCs, failed to improve axonal regeneration (Kuchibhotla et al., 2009; Filous et al., 2014; Anderson et al., 2016). Thus, directly targeting CSPGs would be a better choice than the ablation of particular CSPG-producing cell types for therapeutic interventions to regulate CSPGs.

It is still controversial about the CSPGs effect, however, CSPGs are recognized mostly inhibitory effects and can hinder regeneration of axons across lesions in chronic SCI environment. Chondroitinase ABC (ChABC) is a bacterial enzyme that can effectively degrade CSPGs, including NG2 and was shown to promote functional gains after intrathecal administration in mouse models (Bradbury et al., 2002; Jones et al., 2002). Additional evidence has also shown that combined administration of ChABC with NPCs enhances transplant survival and host axon remyelination (Ikegami et al., 2005; Carter et al., 2011). A more recent study of large-scale CSPG digestion by direct lentiviral ChABC gene delivery into rat spinal cords resulted in a reduced volume of cavitation and enhanced axon preservation. The treated rats also displayed improved sensorimotor function on behavioral and electrophysiological assessments (Bartus et al., 2014). We also reported that ChABC administration reduced chronic injury scar and significantly improved NSCs derived from induced pluripotent stem cell (iPSC-NSC) survival with clear differentiation into all three neuroglial lineages. ChABC pretreatment can ‘unlock’ the chronically injured spinal cord to produce a microenvironment conducive to regenerative iPSC therapy (Suzuki et al., 2017). The optimal delivery modality for exciting therapy with ChABC remains to be elucidated. In the future, chronic SCI research may focus on the exploration of human CNS-specific analogs of ChABC and their development.

## Characteristics of Neural Stem/Neural Progenitor Cells

Neural stem cells are self-renewing, multipotent cells that initially produce the radial glial progenitor cells that generate the neurons and glia of all animal nervous systems during embryonic development (Barnabé-Heider et al., 2010; Beattie and Hippenmeyer, 2017). Some neural progenitor stem cells remain in highly restricted regions of the adult vertebrate brain to produce neurons throughout life (Clarke et al., 2000). NSCs are primarily characterized by their capacity to differentiate into neurons, astrocytes, and oligodendrocytes.

Neural progenitor cells are the progenitor cells of the CNS that produce the glial and neuronal cell types present in the CNS. NPCs do not generate non-neural cells. Rather, NPCs can be generated *in vitro* by the differentiation of embryonic stem cells or iPSCs, which are derived from adult fibroblasts or blood cells (Takahashi and Yamanaka, 2006; Yamanaka, 2012). NPCs may differentiate into neural cells after transplantation into the injured spinal cord to replace lost or damaged cells, provide trophic support, restore connectivity, and facilitate regeneration (Ruff et al., 2012; Suzuki et al., 2017).

Neural stem cells can be derived from various regions along the neuroaxis during embryonic development and in adult life (Salewski et al., 2012). They have been isolated from both the subependymal zone of the adult mammalian brain and the ependymal and non-ependymal regions of adult mammalian spinal cord. Single adult NSCs can be isolated *in vitro* in the presence of growth factors such as epithelial growth factor (EGF) and fibroblast growth factor (FGF) that enable the formation and proliferation of clonally derived free-floating colonies. To promote the differentiation and survival of cellular subpopulations *in vitro*, they can be exposed to bone morphogenetic proteins to produce astrocytes (Salewski et al., 2012); insulin-like growth factor-I, interleukin-1, and neuregulin-1 to generate oligodendrocytes; and neurogenin-2 to produce neurons (Salewski et al., 2012).

In the terminology used here, NSCs are multipotent cells that can self-renew and proliferate without limit to produce progeny cells that terminally differentiate into neurons, astrocytes and oligodendrocytes. The non-stem cell progeny of NSCs are referred to as NPCs. In contrast to NSCs, NPCs have the capacity to proliferate and differentiate into more than one cell type. Thus, they can be unipotent, bipotent, or multipotent (Salewski et al., 2012). Unlike those of a stem cell, distinguishing features of a NPC are its limited proliferative ability and inability of self-renewal.

## Cell-Based Therapies Using Neural Stem/Neural Progenitor Cells in Chronic Spinal Cord Injury

The two main aims of cell-based treatment for chronic SCI are replacing lost or injured cells such as neurons and oligodendrocytes and providing the cells with a microenvironment that supports or enhances the ability of cells within a lesion to provide neuroprotection and promote regeneration. NSCs/NPCs have the ability to replace the lost cells and remyelinate axons at the injury site and also to provide them with supportive growth and neuroprotective factors (Courtine et al., 2008; Lu et al., 2012; Salewski et al., 2012; Ahuja and Fehlings, 2016; Ahuja et al., 2017; Suzuki et al., 2017).

NSCs/NPCs could differentiate into neurons, oligodendrocytes and astrocytes. Grafted NSCs/NPCs survived in chronic SCI lesion, reduced cavity and promoted axonal regrowth (Lu et al., 2012; Salewski et al., 2012; Suzuki et al., 2017). In addition, differentiated oligodendrocytes promoted the remyelination of axons (Salewski et al., 2012). Differentiated motor neurons and interneurons made new neuronal circuits between host and grafted cells to make new synaptic connections (Suzuki et al., 2017). Also differentiated astrocytes supported to provide the new vascularization and supportive growth/neuroprotective factors (Ahuja and Fehlings, 2016; Ahuja et al., 2017).

### PubMed/Medline Search to Identify Experimental and Clinical Studies Describing Treatment of Chronic Spinal Cord Injury With Neural Stem/Neural Progenitor Cells

The PubMed/Medline database was searched in August 2021 [search strategy: “(neural stem cell) OR (neural progenitor cell) AND (chronic SCI)”]. Articles on cell transplantation within 4 weeks after SCI were excluded from the analysis, as were articles on other forms of cell transplantation without NSCs/NPCs and those lacking outcomes describing motor functional recovery. In total, 184 articles were initially identified, and 33 articles were ultimately selected based on the above criteria. There were 10 review articles, 16 basic research articles, and 6 articles on clinical transplantation (Tables 2, 3, 4).

**TABLE 2:** Characteristics of included experimental studies for chronic SCI.

Authors, year	Study title	Timing of cell transplantation	Location of injury	SCI model	Species	Cell therapy	Cell source	Route of administration	Combination	Locomotion test	Result
Tashiro et al., 2016	Functional Recovery from Neural Stem/Progenitor Cell Transplantation Combined with Treadmill Training in Mice with Chronic Spinal Cord Injury	7 Weeks after SCI	Thoracic	Severe contusive injury IH Impactor	Mice	NSCs/NPCs	Fetal brain, Mouse	Transplant into injured spinal cord	Treadmill training	Basso Mouse Scale (BMS) von Frey monofilament test Hargreaves plantar test	Enhanced the recovery of motor and sensory functions
Suzuki et al., 2017	Neural stem cell mediated recovery is enhanced by Chondroitinase ABC pretreatment in chronic cervical spinal cord injury	8 Weeks after SCI	Cervical	Moderate to severe injury Clip compression injury	Mice	NSCs/NPCs	iPSCs, Mouse	Transplant into 1 mm away from injured spinal cord	ChABC	BMS CatWalk digital gait analysis Forelimb grip strength meter Inclined plane test	Motor functional recovery of upper limbs New synaptic formation
Ruzicka et al., 2019	The Effect of iPS-Derived Neural Progenitors Seeded on Laminin-Coated pHEMA-MOETACl Hydrogel with Dual Porosity in a Rat Model of Chronic Spinal Cord Injury	5 Weeks after SCI	Thoracic	Balloon compression injury	Rat	NPCs	iPSCs, Human	Transplant into injured spinal cord	Laminin-coated hydrogel	Basso, Beattie, and Bresnahan (BBB) open field test Plantar test	No significant recovery of locomotor function Reduced cavitation and graft cell survived
Cheng et al., 2016	Local versus distal transplantation of human neural stem cells following chronic spinal cord injury	4 Weeks after SCI	Thoracic	Moderate contusion injury The Multicenter Animal Spinal Cord Injury Study Impactor	Rat	NSCs	iPSCs, Human	Transplant into injured spinal cord Transplant into distally site from SCI Intrathecal	None	BBB open field test	Functional improvement: injected distally to the site of injury
Nori et al., 2018	Human Oligodendrogenic Neural Progenitor Cells Delivered with Chondroitinase ABC Facilitate Functional Repair of Chronic Spinal Cord Injury	7 Weeks after SCI	Thoracic	Moderate to severe injury Clip compression injury	Rat	NSCs/NPCs	Directly reprogrammed NPCs, Human	Intrathecal	ChABC	BBB open field test von Frey monofilament test CatWalk digital gait analysis	Motor functional recovery Remyelination
Salazar et al., 2010	Human neural stem cells differentiate and promote locomotor recovery in an early chronic spinal cord injury NOD-scid mouse model	30 days after SCI	Thoracic	Contusion injury IH device	Mice	NSCs	Fetal brain, Human	Transplant into both rostral and caudal to the injury epicenter	None	BMS CatWalk digital gait analysis von Frey monofilament test	Functional improvement
Nutt et al., 2013	Caudalized human iPSC-derived neural progenitor cells produce neurons and glia but fail to restore function in an early chronic spinal cord injury model	4 Weeks after SCI	Cervical	Contusion injury Fourth generation Ohio State Injury Device	Rat	NPCs	iPSCs, Human	Transplant into injured spinal cord	NeuroRegen scaffold	Limb-use asymmetry test Forelimb reaching task von Frey monofilament test	No significant improvement in forelimb function or induced allodynia
Kusano et al., 2010	Transplanted neural progenitor cells expressing mutant NT3 promote myelination and partial hindlimb recovery in the chronic phase after spinal cord injury	6 Weeks after SCI	Thoracic	Microvascular clip injury	Rat	NPCs secreting Neutrotrophin-3	Fetal brain, Rat	Transplant around the cavity	Neutrotrophin-3	BBB open field test	Enhanced myelin formation Partial improvement of hindlimb function
Karimi-Abdolrezaee et al., 2010	Synergistic effects of transplanted adult neural stem/progenitor cells, chondroitinase, and growth factors promote functional repair and plasticity of the chronically injured spinal cord	6 Weeks after SCI	Thoracic	Clip compression injury	Rat	NSCs/NPCs	Fetal brain, Mouse	Transplant into both rostral and caudal to the injury epicenter	ChABC, EGF, bFGF, PDGF-AA	BBB open field test Grid-walking analysis von Frey monofilament test	Promoted the axonal integrity and plasticity of the corticospinal tract and enhanced the plasticity of descending serotonergic pathways.
Dagci et al., 2009	Alterations in the expression of the apurinic/apyrimidinic endonuclease-1/redox factor-1 (APE/ref-1) and DNA damage in the caudal region of acute and chronic spinal cord injured rats treated by embryonic neural stem cells	4 Weeks after SCI	Thoracic	Selectively ablated only the lateral white matter tracts and a minimal portion of the dorsal and ventral gray matter.	Rat	NSCs	Embryo, Rat	Transplant into injured spinal cord	None	BBB open field test	Decreased DNA damage levels
Pfeifer et al., 2006	Autologous adult rodent neural progenitor cell transplantation represents a feasible strategy to promote structural repair in the chronically injured spinal cord	8 Weeks after SCI	Cervical	Dorsal corticospinal tract were transected using a tungsten wire knife	Rat	Autologous NPCs	Adult Brain, Rat	Transplant into injured spinal cord	Fibroblasts	None	Promoted axon regrowth and tissue replacement in SCI
Jones et al., 2021	Human Embryonic Stem Cell-derived Neural Crest Cells Promote Sprouting and Motor Recovery Following Spinal Cord Injury in Adult Rats	7 Weeks after SCI	Cervical	Lateral funiculus and adjacent gray matter were transected	Rat	Neural crest cells	ES cells	Transplant into injured spinal cord	None	Vertical cylinder test	Promoted remodeling of descending raphespinal projections and contributed to the partial recovery of forelimb motor function
Kumamaru et al., 2013	Therapeutic activities of engrafted neural stem/precursor cells are not dormant in the chronically injured spinal cord	12 Weeks after SCI	Thoracic	Moderate contusion injury Infinite Horizons Impactor Precision Systems Instrumentation	Mice	NSCs/NPCs	Embryo, Mouse	Transplant into both rostral and caudal to the injury epicenter	None	BMS Grip walk test Footprint analysis	No significant recovery of locomotor function Differentiated into neurons/ oligodendrocytes
Okubo et al., 2018	Treatment with a Gamma-Secretase Inhibitor Promotes Functional Recovery in Human iPSC- Derived Transplants for Chronic Spinal Cord Injury	6 Weeks after SCI	Thoracic	Contusive injury IH impactor	Mice	NSCs/NPCs	iPSCs, Human	Transplant into injured spinal cord	Gamma-secretase inhibitor	BMS Rotarod testing Treadmill gait analysis	Promoted and maintained motor function recovery Induced red myelination and promoted axonal regeneration
Xu et al., 2021	Transplantation of Human Neural Precursor Cells Reverses Syrinx Growth in a Rat Model of Post-Traumatic Syringomyelia	10 Weeks after SCI	Thoracic	IH spinal cord impactor	Rat	NSCs/NPCs	iPSCs, Human	Transplant into injured spinal cord	Neuroepithelial-like stem cells	BBB open field test KSAT for swim performance Beam walk assessing the ability to traverse narrow square beams Grid walk counting misplaced steps	Reduced cyst volumes
Martín-López et al., 2021	Modeling chronic cervical spinal cord injury in aged rats for cell therapy studies	4 Weeks after SCI	Cervical	Contusion injury Fourth generation Ohio State Injury Device	Rat	NPCs	iPSCs, Human	Transplant into injured spinal cord	None	BBB open field test	Grafted cells survived and did not cause tumors

**TABLE 3:** Characteristics of included clinical trials for chronic SCI.

Authors, year	Study title	Timing of cell transplantation	Location of injury	Cell therapy	Cell source	Route of administration	Combination	Result
Curtis et al., 2018	A First-in-Human, Phase I Study of Neural Stem Cell Transplantation for Chronic Spinal Cord Injury	Chronic	Th2-T12	NSCs (NSI-566)	Human spinal cord	Transplant into injured spinal cord		NSI-566 transplanted in the spinal injury site of patients can be performed safely.
Levi et al., 2019	Clinical Outcomes from a Multi-Center Study of Human Neural Stem Cell Transplantation in Chronic Cervical Spinal Cord Injury	Chronic (4–24 months)	C5-7	NSCs (HuCNS-SC)^®^	Human brain	Transplant into injured spinal cord Intramedullary free-hand (manual) transplantation		Cohorts I and II demonstrated a trend toward Upper Extremity Motor Score (UEMS) and Graded Redefined Assessment of Strength, Sensibility, and Prehension (GRASSP) motor gains in the treated participants.
Levi et al., 2018	Emerging Safety of Intramedullary Transplantation of Human Neural Stem Cells in Chronic Cervical and Thoracic Spinal Cord Injury	Chronic	C5-7 Th2-T12	NSCs (HuCNS-SC)^®^	Human brain	Transplant into injured spinal cord Intramedullary free-hand (manual) transplantation		A manual injection technique are safe and feasible.
Ghobrial et al., 2017	Human Neural Stem Cell Transplantation in Chronic Cervical Spinal Cord Injury: Functional Outcomes at 12 Months in a Phase II Clinical Trial	Chronic	Cervical/ Thoracic	NSCs (HuCNS-SC)^®^	Human brain	Transplant into injured spinal cord Intramedullary free-hand (manual) transplantation		Improvements in overall mean functional outcomes measures.
Moviglia et al., 2009	Case report on the clinical results of a combined cellular therapy for chronic spinal cord injured patients	Chronic	Cervical/ Thoracic	Autologous NSCs		Feeding artery infusion	Bone marrow mononuclear cells Effector T cells	Five of eight patients evolved from ASIA A to ASIA D.
Moviglia et al., 2006	Combined protocol of cell therapy for chronic spinal cord injury. Report on the electrical and functional recovery of two patients	Chronic	Cervical/ Thoracic	Autologous NSCs	BMSCs	Feeding artery infusion	Neurorehabilitation	Effective for the repair of chronic SCI.

**TABLE 4:** Included review articles on chronic SCI.

Authors, year	Study title
Yousefifard et al., 2016	Neural stem/progenitor cell transplantation for spinal cord injury treatment; A systematic review and meta-analysis
Platt et al., 2020	Stem Cell Clinical Trials in Spinal Cord Injury: A Brief Review of Studies in the United States
Jin et al., 2016	Transplantation of neural progenitor cells in chronic spinal cord injury
Vancamp et al., 2020	Thyroid Hormone and Neural Stem Cells: Repair Potential Following Brain and Spinal Cord Injury
Lane et al., 2017	Improving the therapeutic efficacy of neural progenitor cell transplantation following spinal cord injury
Curt, 2012	Human neural stem cells in chronic spinal cord injury
Tsuji et al., 2019	Concise Review: Laying the Groundwork for a First-In-Human Study of an Induced Pluripotent Stem Cell-Based Intervention for Spinal Cord Injury
Dalamagkas et al., 2018	Translational Regenerative Therapies for Chronic Spinal Cord Injury
Nagoshi et al., 2020	Regenerative therapy for spinal cord injury using iPSC technology
Suzuki and Sakai, 2021	Current Concepts of Stem Cell Therapy for Chronic Spinal Cord Injury

### Neural Stem/Neural Progenitor Cells Treatment for Chronic Spinal Cord Injury in Experimental Models

We show the characteristics of the included experimental studies for chronic SCI in Table 2. Several articles reported on the neurological changes occurring following the transplantation of NSCs/NPCs only in rat and mice models of chronic SCI (Kusano et al., 2010; Salazar et al., 2010; Cheng et al., 2016; Tashiro et al., 2016; Suzuki et al., 2017; Nori et al., 2018; Okubo et al., 2018; Jones et al., 2021). However, the other articles revealed no significant recovery of locomotor function (Kumamaru et al., 2013; Nutt et al., 2013; Ruzicka et al., 2019; Martín-López et al., 2021).

The combinatory and synergic effects of other treatments with NSCs/NPCs transplantation were also reported. Rehabilitation was the most common combinatory treatment used clinically. Treadmill exercise combined with NPC transplantation was found to promote neuronal differentiation and regeneration and maturation of neural circuits. Further, it enhanced the recovery of motor and sensory functions even when the intervention took place during the chronic phase (Tashiro et al., 2016). Several previous articles reported on the use of a scaffold with cell transplantation: laminin-coated hydrogel and the NeuroRegen scaffold (Nutt et al., 2013; Ruzicka et al., 2019). Combinatory scaffold use reduced cavitation and supported graft-cell survival (Ruzicka et al., 2019).

Some papers revealed that NSCs/NPCs grafts reduced cyst volume and promoted axon regrowth through the synergic effect of combining cell grafts with fibroblasts or neuroepithelial-like stem cells (Pfeifer et al., 2006; Xu et al., 2021).

Synergic treatment with the neurotrophic factors EGF, bFGF, PDGF-AA, and NT-3 along with NSCs/NPCs transplantation was also reported and led to partial improvement of hindlimb function (Karimi-Abdolrezaee et al., 2010; Kusano et al., 2010).

The most promising combinatory treatment reported in these articles was the injection of ChABC prior to NSCs/NPCs transplantation. ChABC pretreatment work to ‘unlock’ the chronically injured spinal cord to produce a microenvironment conducive to regenerative NSC/NPC therapy (Karimi-Abdolrezaee et al., 2010; Suzuki et al., 2017; Nori et al., 2018). In addition, all three of these papers reported partial motor functional recovery following ChABC and NSCs/NPCs treatment.

One article revealed that the sites of cell injection were quite important to regenerate damaged spinal cord (Cheng et al., 2016). Among the articles we reviewed that reported neurologically functional recovery, all reported transplantation rostral and/or distal to the site of the SCI epicenter (Kusano et al., 2010; Salazar et al., 2010; Cheng et al., 2016; Suzuki et al., 2017; Nori et al., 2018; Okubo et al., 2018; Jones et al., 2021).

These articles indicate that NSCs/NPCs injection sites and the synergic effects of ChABC and neurotrophic factors are important factors leading to motor functional recovery following chronic SCI as a combinatory treatment with NSCs/NPCs transplantation. Even if only NSCs/NPCs transplantation is performed, it can lead to and support histological regeneration occurring at the site of chronic SCI.

### Neural Stem/Neural Progenitor Cells Treatment for Chronic Spinal Cord Injury in Clinical Studies

We show the characteristics of the included clinical studies for chronic SCI in Table 3. The transplanted NSCs/NPCs were autologous NSI-566 and HuCNS-SC^®^ cells (Moviglia et al., 2006, 2009; Ghobrial et al., 2017; Curtis et al., 2018; Levi et al., 2018, 2019). Cell sources were bone marrow mesenchymal stem cells (MSCs) and cells from the human spinal cord and brain. All of the articles revealed that transplantation of NSCs/NPCs in the site of the patients’ SCI can be performed safely. In addition, injection techniques including free-hand transplantation and infusion in the feeding artery were safe and feasible. One case report showed that five of eight patients evolved from ASIA (American Spinal Injury Association) class A to ASIA D (Moviglia et al., 2009). Injection of HuCNS-SC^®^ cells was also reported to lead to improvement in overall mean functional outcome measures.

However, several critical points are still remain about the NSCs/NPCs source, safety, administration route and the optimal time-window of efficacy in clinical (Tsuji et al., 2019; Nagoshi et al., 2020; Suzuki and Sakai, 2021; Table 5). Ethical concerns still remain the use of NSCs/NPCs harvested from fetal or embryonic stem cells (Nagoshi et al., 2020). iPSCs is one of the ideal NSCs/NPCs source, however, still have the genetic and epigenetic abnormalities and subsequent tumorigenicity (Nagoshi et al., 2020). We have several administration route for cell grafts, intramedullary, intrathecal, intraventricular and intravascular. Each administration still have the advantages and disadvantages in clinical (Yamazaki et al., 2020). Many researchers have investigated the phases to determine the optimal time-window of efficacy for NSCs/NPCs therapy in animals (Tetzlaff et al., 2011). For grafted cell survival, the microenvironment in chronic phase was the most difficult one. However, the clinical study does not necessarily require double-arm study (Oh et al., 2016). Several complications following stem cell grafts in clinical were reported that transient neuropathic pain, transient deterioration in sensorimotor symptoms, subarachnoid hemorrhage, cerebrospinal fluid leakage, subcutaneous seroma, fever, transient hypertension, vomiting, urinary tract infection, abnormal blood profiles, pulmonary thromboembolism and general body ache (Jeong et al., 2020).

**TABLE 5:** Promising attempts, limitations and discussing points in NSC/NPC treatment for chronic SCI.

Promising attempts	References	Limitations/ Discussion points	References
Improvement of functional outcome	Moviglia et al., 2009; Kusano et al., 2010; Karimi-Abdolrezaee et al., 2010; Salazar et al., 2010; Cheng et al., 2016; Tashiro et al., 2016; Suzuki et al., 2017; Nori et al., 2018; Okubo et al., 2018; Jones et al., 2021	Partial functional recovery	Tetzlaff et al., 2011; Oh et al., 2016; Anderson et al., 2017; Lane et al., 2017; Deng et al., 2018; Tsuji et al., 2019; Yamazaki et al., 2020; Nagoshi et al., 2020; Suzuki and Sakai, 2021
Grafted NSC/NPC survive in chronic SCI lesion		Partial control of directing cell differentiation	
Differentiate into neuronal lineages -Neuron, Oligodendrocyte and Astrocyte-		Limitation of functional integration with the host neural circuitry	
Promotion of axon regrowth		Variability in both anatomical and functional outcomes	
Reduction of cyst volume		Safety in clinical trials (NSC/NPC source)	
Promotion of Neural Pathway Plasticity	Pfeifer et al., 2006; Ruzicka et al., 2019; Xu et al., 2021	Lack of control on the processes rewiring the neural new circuits.	
Remyelination		Administration route -Intramedullary, Intrathecal, Intraventricular and Intravascular-	
		Optimal time-window of efficacy	
		Issue of cost-benefit ratio	

There are still additional limitations of NSCs/NPCs treatment for chronic SCI in clinical studies. We could have only partial functional recovery and there were variability in both anatomical and functional outcomes in several articles (Lane et al., 2017; Table 5). For example, preclinical trials using HuCNS-SC^®^ cells revealed no evidence of efficacy (Anderson et al., 2017). They mentioned that the data raised questions about the development and validation of potency/comparability assays for clinical testing of cell products.

Several critical points are still remain, however, NSCs/NPCs treatment has the promising attempts in treatment for chronic SCI from the many kinds of preclinical trials (Kusano et al., 2010; Salazar et al., 2010; Kumamaru et al., 2013; Nutt et al., 2013; Cheng et al., 2016; Tashiro et al., 2016; Suzuki et al., 2017; Nori et al., 2018; Okubo et al., 2018; Ruzicka et al., 2019; Jones et al., 2021; Table 5). Grafted NSCs/NPCs survived in chronic SCI lesion and differentiated into neuronal lineages. The cell grafts also reduced cyst volume and promoted axon regrowth, remyelination and neural pathway plasticity. In addition, these therapeutic effects led to pathophysiological regeneration and motor/sensory functional recovery.

### Review Articles on Neural Stem/Neural Progenitor Cells Treatment for Chronic Spinal Cord Injury

We list the review articles on NSCs/NPCs treatment for chronic SCI in Table 4. Many therapeutic approaches have been reviewed in these articles in the attempt to treat chronic SCI, and many studies reported that cellular transplantation offered the greatest promise in reconstituting the architecture of the damaged spinal cord (McDonald et al., 1999; Curt, 2012; Jin et al., 2016; Yousefifard et al., 2016; Lane et al., 2017; Dalamagkas et al., 2018; Tsuji et al., 2019; Nagoshi et al., 2020; Platt et al., 2020; Vancamp et al., 2020; Suzuki and Sakai, 2021). Most of the ongoing clinical trials are targeting acute to subacute SCI; however, several are including the chronic phase as well (Table 4). Some of the articles showed that the grafted NSCs/NPCs had a strong capacity to differentiate into neural cells and that they retained the secretory function of growth factors and regenerative molecules. However, two articles pointed out that the recovery of locomotor function was quite difficult following the transplantation of NSCs/NPCs only (Nagoshi et al., 2020; Suzuki and Sakai, 2021). Several review articles mentioned that combinatory therapy could be an appropriate strategy with the use of drug administration and rehabilitation, in addition to NSCs/NPCs transplantation in chronic SCI (Tsuji et al., 2019; Nagoshi et al., 2020; Suzuki and Sakai, 2021).

Some papers reviewed safety issues and their resolution for NSCs/NPCs derived from human iPSCs (Tsuji et al., 2019; Nagoshi et al., 2020; Table 5). The primary safety issue of concern was the risk of tumor formation (Tsuji et al., 2019; Nagoshi et al., 2020). One article listed five key issues involved in improving the safety of NSCs/NPCs derived from human iPSCs. First, not to use genetically unstable human iPSC; second, to prevent contamination by undifferentiated pluripotent cells; third, to prevent the transformation of progenitor cells into tumor; fourth, to minimize the risk of proliferation of differentiation-resistant abnormal cells; and fifth, to remove any abnormal cells after the transplantation (Deng et al., 2018; Tsuji et al., 2019).

### Neurorehabilitation in Experimental and Clinical Studies Following Neural Stem/Neural Progenitor Cells Therapies for Chronic Spinal Cord Injury

We described about SCI rehabilitation in clinical studies on stem cell therapies using mesenchymal stem cells and olfactory ensheathing cells (OECs) in previous article (Suzuki and Sakai, 2021). However, only the article reported the combinatory treatment with treadmill training and NSCs/NPCs graft in chronic SCI in mice (Tashiro et al., 2016; Table 2). This article reported that treadmill training was started just after NSCs/NPCs transplantation. In addition, they revealed that the combined therapy enhanced these independent effects of each single therapy (Tashiro et al., 2016). In addition, only one article mentioned about the combinatory treatment with neurorehabilitation and NSCs/NPCs therapy in chronic SCI in clinical study (Curtis et al., 2018; Table 3). This clinical study reported only case reports following NSCs/NPCs graft, the rehabilitation was not same in each patient and not systematic neurorehabilitation program. Therefore, it is difficult to discuss about the efficacy of neurorehabilitation following NSCs/NPCs treatment in chronic SCI.

In this session we would like to discuss about the timing of rehabilitation and what kinds of rehabilitation were combined with several stem cell therapies in chronic SCI. In the transplantation of OECs, it was reported that the quality and quantity of rehabilitation influenced the long-term outcome in patients with chronic SCI (Huang et al., 2012). However, it was not describing about the timing of rehabilitation and what type of rehabilitation was performed (Huang et al., 2012). To our knowledge, unfortunately, this is the only study investigating the relationship between functional recoveries and the sufficient/insufficient of neurorehabilitation following stem cell treatment in chronic SCI patients (Tashiro et al., 2021). There were several combinatory treatment of stem cell therapy (Umbilical cord blood cell and BMSC) and neurorehabilitation in ongoing clinical trials (NCT03979742, NCT01354483, and NCT01393977, see footnote 1). However these clinical trials are ongoing, therefore, these data are not published now.

## Ongoing Clinical Trials Currently Targeting Chronic Spinal Cord Injury that Use Neural Stem/Neural Progenitor Cells

We list the ongoing clinical trials currently targeting chronic SCI that use NSCs/NPCs in Table 6 (see footnote 1 [accessed 20 August 2021]). All of the ongoing clinical trials were started on the basis of the good results obtained in preclinical studies. In chronic SCI, phase 1 and 2 studies are now ongoing to transplant NSCs/NPCs using autologous fresh stem cells containing product or MSCs. Only the NCT02688049 study is transplanting the NeuroRegen scaffold with NSCs/NPCs.

**TABLE 6:** Ongoing clinical trials currently targeting chronic SCI utilizing NSC/NPCs (https://www.clinicaltrials.gov/).

Identifier	Study title	Phase	Subjects (participants)	Cell therapy	Route of administration	Combination
NCT04205019	Safety Stem Cells in Spinal Cord Injury	Phase 1	10	Neuro-Cells (autologous fresh stem cell-containing product)	Intrathecal	
NCT02688049	NeuroRegen Scaffold*™* Combined With Stem Cells for Chronic Spinal Cord Injury Repair	Phase 1 Phase 2	30	NSCs with mesenchymal stem cells	Transplant into injured spinal cord	NeuroRegen scaffold
NCT01772810	Safety Study of Human Spinal Cord-derived Neural Stem Cell Transplantation for the Treatment of Chronic SCI	Phase 1	8	Human spinal cord-derived NSCs	Surgical implantation	

The NCT04205019 phase 1 clinical study begun on November 14, 2020, is an open clinical trial designed to investigate the safety of the intrathecal application of Neuro-Cells in the treatment of patients with end-stage (i.e., chronic) traumatic complete (AISA grade A) or incomplete (AISA grade B/C) SCI.

In the NCT02688049 phase 2 clinical study, patients with chronic SCI (ASIA grade A) are receiving NeuroRegen Scaffold with 10 million NSCs transplanted after localized scarring is cleared and after surgery patients undergo comprehensive rehabilitation, psychological, and nutritional measures. This clinical trial was started in January 2016.

The NCT01772810 trial is a phase 1 clinical study of SCI injury classified as AISA A in the UCSD Medical Center, Division of Neurosurgery that initially started in August 2014. The treatment is surgical implantation of human spinal cord-derived NSCs. The inclusion criterion is at least 1 year but no more than 2 years from time of injury to the time of surgery.

## Future Candidates for Combinatory Treatment With Neural Stem/Neural Progenitor Cells Transplantation for Chronic Spinal Cord Injury

As previously mentioned, combinatory treatment with NSCs/NPCs is an important factor leading to improved recovery of locomotor function in chronic SCI. Several other approaches were reported in the treatment of chronic SCI, and we reviewed rehabilitation and scaffold treatment for chronic SCI as the most promising candidates for combinatory treatment with NSCs/NPCs.

### Tissue Engineering Approaches for Chronic Spinal Cord Injury

Several scaffolds were reported for use in bridging defects in experimental models of chronic SCI (Austin et al., 2012; Haggerty and Oudega, 2013; Pawar et al., 2015; Chedly et al., 2017; Koffler et al., 2019). It was reported that anisotropic alginate hydrogel scaffolds promoted axonal growth across chronic spinal cord transections after scarring was removed (Huang et al., 2020). Both electrophysiological conductivity and locomotor function improved significantly after engraftment with this scaffold. Transplantation of human umbilical cord-derived MSCs seeded in collagen scaffolds reduced scar formation and promoted functional recovery in chronic SCI (Li et al., 2017; Wang et al., 2018). Some articles revealed the efficacy of Laminin-Coated pHEMA-MOETACl Hydrogel (Ruzicka et al., 2019), HPMA-RGD hydrogels (Hejcl et al., 2010), and chimeric self-assembling nanofiber (Tavakol et al., 2016). However, these were combined with iPSC-derived NPCs or MSCs. Three-dimensional aligned nanofiber-hydrogel scaffolds (Nguyen et al., 2017), self-assembling scaffolds, Taxol-modified collagen scaffolds (Yin et al., 2021), graphene oxide scaffolds (López-Dolado et al., 2016), and nanostructured composite scaffolds (Gelain et al., 2011) were reported for the treatment of chronic SCI. These articles revealed the possibility of recreating an anatomical, structural, and histological framework that could lead to the replacement of large hollow tissue gaps in the chronically injured spinal cord, thus encouraging axonal regeneration and neurological recovery.

In a clinical study, peripheral nerve grafts combined with a chitosan-laminin scaffold were grafted in chronic SCI patients and were reported to enhance regeneration (Amr et al., 2014). The NeuroRegen scaffold was also reported to be transplanted into 51 chronic complete SCI patients, resulting in 16 patients achieving expansion of their sensation level and 30 patients experiencing enhanced reflexive defecation sensation or increased skin sweating below the injury site. Nearly half of the patients with chronic cervical SCI developed enhanced finger activity (Tang et al., 2021). The study also revealed that increased finger activity, enhanced trunk stability, defecation sensation, and recovery of autonomic neural function were observed in some patients following transplantation of the NeuroRegen scaffold combined with human MSCs (Zhao et al., 2017).

### Rehabilitation Approaches for Chronic Spinal Cord Injury

In this section, we review the articles that mention rehabilitative training after chronic SCI in clinical studies. We focus on robotic-assisted gait training (RAGT) and functional electrical stimulation (FES) for enhancing the recovery of neuronal plasticity as new rehabilitation approaches.

One of the challenges in neurorehabilitation targeting the restoration of functional independence and quality of life is recovery of the ability to plan and execute movement again (Maier et al., 2019). Several researchers found that RAGT in SCI patients improved the cardiorespiratory, urinary, musculoskeletal, neuronal and somatosensory systems, due to body compensation and neural plasticity (Fleerkotte et al., 2014; Labruyère and van Hedel, 2014; Holanda et al., 2017; Nam et al., 2017). A review article that included 10 trials involving 502 participants showed that the acute RAGT groups showed significantly greater improvements in gait distance, leg strength, and functional level of mobility and independence than the over-ground training groups. Significantly greater improvements in speed and balance were also observed in the chronic RAGT group versus the group with no intervention (Nam et al., 2017). Another systematic review showed that significant progress was being made with robotic devices as an innovative and effective therapy for the rehabilitation of individuals with SCI (Holanda et al., 2017). However, Piira et al. (2019) reported in a clinical trial that RAGT did not improve walking function in patients with chronic incomplete SCI. Wearable powered robotic exoskeletons allow chronic complete SCI patients to perform over-ground walking. In addition, different exoskeleton software control of the smoothness of the gait pattern improves functional outcome, eliminating the relationship between anthropometric factors and gait performance (Guanziroli et al., 2019).

Some other papers revealed that neurorehabilitation using a voluntary driven exoskeletal (VDE) robot improved trunk function and voluntary contractions (Grasmücke et al., 2017; Okawara et al., 2022). VDE training immediately improved lower limb function and muscle activity and correct synergy control of the lower limb muscles during gait and also increased excitability in the primary somatosensory cortex (Sczesny-Kaiser et al., 2015; Shimizu et al., 2017; Matsuda et al., 2018; Tan et al., 2018).

Recently, gait training using the Alternating Hybrid Assistive Limb (HAL^®^) Robot was reported. By combining gait training using HAL-assisted and conventional gait training with physical therapy, the ability of patients with a chronic SCI to walk may be improved over a short period (Sczesny-Kaiser et al., 2015; Shimizu et al., 2017; Matsuda et al., 2018; Kanazawa et al., 2019). The potential for gait training using HAL to improve the ability of patients with chronic severe incomplete tetraplegic SCI to walk was also shown. HAL motion-assistive technologies contributed to improvement in patient walking ability by facilitating proper joint motion and loading and unloading muscle movements (Soma et al., 2021).

A systematic review of the clinical benefits of rehabilitation training in SCI reported that robotic-assisted treadmill training improved lower extremity function (95% CI 3.44, 6.56) compared with related controls, and FES also significantly increased upper extremity independence (95% CI 0.37, 5.48) (Duan et al., 2021).

FES treatment is one of the new challenges in active rehabilitation training for chronic SCI patients (Marquez-Chin and Popovic, 2020). Several clinical studies reported the efficacy of FES therapy for chronic SCI (Bajd et al., 1999; Popovic et al., 1999, 2011; Mangold et al., 2005; Thrasher et al., 2006; Kapadia et al., 2013, 2014). These articles reported increases in strength and improvement in drop foot and plantar flexion after training using a neuroprosthesis for walking after SCI (Bajd et al., 1999). The efficacy of FES treatment to restore the ability to walk following chronic SCI revealed significantly greater improvement in locomotion function with FES treatment compared with a non-FES treatment-controlled intervention (Thrasher et al., 2006). In one recent study, a phase I randomized control trial was conducted in the same population (chronic incomplete SCI between C2 and T12 levels) (Kapadia et al., 2014). The efficacy of 6 months use of the Bionic Glove was also shown to improve upper limb function (increased power grasp and/or range of movements) in individuals with tetraplegia resulting from SCI at the C5–C7 level (Popovic et al., 1999). Several studies reported improvements in grasping function or muscle strength in the majority of 11 individuals who received FES training using a neuroprosthesis for grasping (Popovic et al., 1999; Mangold et al., 2005; Kapadia et al., 2013).

## Conclusion

Currently, numerous clinical and experimental studies have shown positive results in terms of functional improvement with neural stem/progenitor cell treatment in chronic SCI. There are still some inherent limitations in human chronic SCI trials. However, promising results have been reported in basis research and clinical trials. We are convinced that neural stem/progenitor cell therapy will provide the drastic treatment needed for chronic SCI patients in the near future.

## Author Contributions

HS and TS designed the review outline. HS, MF, TS, and NN drafted the manuscript. YI and MF provided critical review of the manuscript. All authors have read and agreed to the published version of the manuscript.

## Conflict of Interest

The authors declare that the research was conducted in the absence of any commercial or financial relationships that could be construed as a potential conflict of interest.

## Publisher’s Note

All claims expressed in this article are solely those of the authors and do not necessarily represent those of their affiliated organizations, or those of the publisher, the editors and the reviewers. Any product that may be evaluated in this article, or claim that may be made by its manufacturer, is not guaranteed or endorsed by the publisher.

## Footnotes

1
https://www.clinicaltrials.gov
